# Rapid, scalable and highly automated HLA genotyping using next-generation sequencing: a transition from research to diagnostics

**DOI:** 10.1186/1471-2164-14-221

**Published:** 2013-04-04

**Authors:** Martin Danzer, Norbert Niklas, Stephanie Stabentheiner, Katja Hofer, Johannes Pröll, Christina Stückler, Edeltraud Raml, Helene Polin, Christian Gabriel

**Affiliations:** 1 Department of Immunogenetics, Red Cross Transfusion Service for Upper Austria, Krankenhausstraße 7, Linz 4017, Austria; 2 Hamilton Robotics GmbH, Fraunhoferstraße 17, Martinsried 82152, Germany

## Abstract

**Background:**

Human leukocyte antigen matching at allelic resolution is proven clinically significant in hematopoietic stem cell transplantation, lowering the risk of graft-versus-host disease and mortality. However, due to the ever growing HLA allele database, tissue typing laboratories face substantial challenges. In light of the complexity and the high degree of allelic diversity, it has become increasingly difficult to define the classical transplantation antigens at high-resolution by using well-tried methods. Thus, next-generation sequencing is entering into diagnostic laboratories at the perfect time and serving as a promising tool to overcome intrinsic HLA typing problems. Therefore, we have developed and validated a scalable automated HLA class I and class II typing approach suitable for diagnostic use.

**Results:**

A validation panel of 173 clinical and proficiency testing samples was analysed, demonstrating 100% concordance to the reference method. From a total of 1,273 loci we were able to generate 1,241 (97.3%) initial successful typings. The mean ambiguity reduction for the analysed loci was 93.5%. Allele assignment including intronic sequences showed an improved resolution (99.2%) of non-expressed HLA alleles.

**Conclusion:**

We provide a powerful HLA typing protocol offering a short turnaround time of only two days, a fully integrated workflow and most importantly a high degree of typing reliability. The presented automated assay is flexible and can be scaled by specific primer compilations and the use of different 454 sequencing systems. The workflow was successfully validated according to the policies of the European Federation for Immunogenetics. Next-generation sequencing seems to become one of the new methods in the field of Histocompatibility.

## Background

Haematopoietic stem cell transplantation (HSCT) is a well defined treatment option for haematological disorders, such as lymphoma, leukaemia and anemia. The outcome depends strongly on the development of graft-versus-host disease [1] next to disease relapse and infection. During the last decades, refinements in clinical practice [2,3] and especially advances in human leukocyte antigen (HLA) matching [4] have steadily improved survival after transplantation by reducing transplant-related complications. Recently published data from comprehensive studies reviewed by Shaw B. E. and colleagues [5] demonstrated a significant survival advantage in HSCT when using donors matched for HLA-A, -B, -C and -DRB1 at allelic resolution. The loci HLA-DQB1 and -DPB1 are still under discussion [6].

In recent years, patients undergoing allogeneic HSCT have greatly benefited from the deeper understanding of the HLA system and notably from high-resolution definition of the classical transplantation antigens. The extensive allelic diversity of these loci is mainly driven by recombination, which results in patchwork pattern of sequence motifs and makes HLA typing at 4-digit and now even at 2-digit level challenging. In this context, the numerous technological advancements in the field of Immunogenetics are of particular importance, eminently the application of DNA-based methodologies [7].

Currently, the “gold standard” for high-resolution HLA typing is Sanger sequencing. Nevertheless, due to the ever increasing numbers of HLA alleles in the International ImMunoGeneTics (IMGT) HLA database [8] (release 3.9.0 includes 8,159 alleles) [9], histocompatibility testing is getting complex even if sequence-based typing (SBT) is used. Defining the phase of sequence motifs becomes more difficult and therefore genotype ambiguity increases exponentially through every new database release [10]. Therefore new typing strategies such as group-specific PCR [11] or allele separation by group-specific sequencing primers [12] prior to sequencing were developed. Though these techniques achieve haplotype separation and are well-established in HLA typing laboratories, they remain labor-intensive and commercial manufacturers are especially unwilling to develop the products in a timely fashion.

In 2004, massively parallel clonal sequencing technologies commonly termed as next-generation sequencing (NGS) techniques [13,14] were introduced with the expectation of trouble-free HLA genotyping without limitations on high-resolution and high-throughput. In 2009 our group [15], independently with H. A. Erlich’s group [16], demonstrated the feasibility of HLA typing using 454 sequencing for the first time. A double blinded multi-center study based on exon amplification by fusion primers was conducted and published in 2011 [17]. This joint study revealed that while 454 sequencing allows for reliable HLA genotype identification, it is a time-consuming, labor-intensive workflow requiring automation. In addition, a systematic algorithm for HLA class I typing was made available using different PCR-based barcoding methods [18]. Most recently, Lank S. M. and colleagues [19,20] provided a cDNA protocol for HLA genotyping with a reduced complexity concerning library preparation. And finally, in contrast to amplicon sequencing, it has been demonstrated that shotgun sequencing using long-range PCR products, fragmentation and multiplex identifier (MID) ligation is a feasible way to define the entire gene sequence of the HLA molecules [21-23].

Nevertheless, the published protocols provide further evidence that next-generation sequencing is a difficult hand-to-use technology and seems inapplicable for laboratories working in the field of Histocompatibility and Immunogenetics. There are unresolved technical issues concerning high workloads for library preparation and overlong processing times. Importantly, bioinformatics at the back-end of the NGS workflow rarely generate a readable HLA typing report, however, an indispensable prerequisite from a medical point of view.

With this in mind, we developed an automated high-resolution HLA typing approach suitable for diagnostic use applying a 454 GS Junior, capturing 17 exons of HLA-A, -B, -C, -DQB1, -DPB1, -DRB1, -DRB3, -DRB4 and -DRB5 (DRB3/4/5). In conclusion, the system was validated extensively under routine conditions in accordance with established policies and procedures (http://www.efiweb.eu) of the European Federation for Immunogenetics (EFI).

## Results

### Sequencing performance

The validation panel consisted of 173 samples, analysed in 18 GS Junior runs. The average raw well count for all runs, defined as PicoTiterPlate (PTP) wells with measurable signal intensity during a sequencing run, was 182,565 ± 32.7. During the validation study we obtained an average passed filter read count of 66,078 ± 16.8 per run with a median read length of 425 bp ± 24. In total, 77.1% ± 4.0 of the internal control sequences had no sequencing error over a length of 400 bp with 98% accuracy. Bead enrichment with the robotic enrichment module (REMe) was stable with enrichment rates for the short pool of 5.7% ± 2.4 and 9.0% ± 4.5 for the pool with long amplicons. Mixed rates, indicating a non-clonal emPCR with beads carrying more than one DNA copy, were measured with 8.5% ± 4.0 (Table 1).

**Table 1:** Sequencing metrics generated during the validation study

**Run**	**Raw wells**	**Passed filter reads**	**# mixed**	**# dot**	**Qual 98% 400 bp**	**Median read length**	**Avg read length**	**Sequenced bases**	**Enrichment pool 1**	**Enrichment pool 2**
	[wells]	[reads]	[reads]	[reads]	[%]	[bp]	[bp]	[Mb]	[%]	[%]
1	196741	40525	45959	5914	70.4	433	419	18	5.7	6.2
2	185532	55284	21285	6652	76.4	439	423	24	11.0	19.2
3	200418	63876	17878	5362	76.1	490	444	31	3.7	8.5
4	180325	58140	12138	5111	79.0	438	420	25	5.9	10.4
5	203507	47888	16014	5627	77.8	437	421	21	4.7	4.9
6	191236	65820	15161	6485	74.5	439	432	29	4.5	8.4
7	195038	84893	14591	6918	76.8	472	436	40	5.7	8.9
8	169943	63738	7626	3978	71.7	471	422	30	2.2	4.8
9	166860	68281	9837	3495	78.4	472	439	32	6.0	8.3
10	181827	76641	11098	5357	76.5	439	424	34	5.3	11.1
11	78145	37859	3424	1574	81.8	407	376	15	7.6	5.8
12	179605	79050	12011	4334	81.7	494	467	39	2.7	7.2
13	152786	49926	11640	4714	70.8	418	406	21	3.4	5.8
14	185278	95070	10123	4410	83.8	476	443	45	4.8	5.8
15	223279	94856	17760	6475	78.4	469	441	44	4.6	8.9
16	166811	75942	12852	4022	76.1	439	428	33	6.0	8.1
17	194555	75140	14330	6253	74.0	468	431	35	4.5	5.5
18	234277	56467	30603	9823	84.1	441	426	25	4.8	6.0

**Figure 1:**
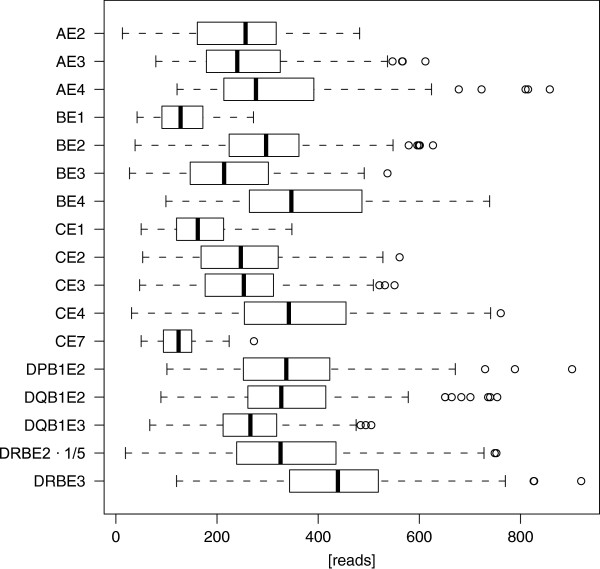
**Raw amplicon read count statistics.** Boxplots show raw amplicon read count for 17 amplicons. For plotting, DRB exon 2 readcounts were downscaled (1/5) for an improved presentation. Readcounts for amplicons without amplification are not included. All amplicons together exhibit a mean of 282 ± 143.

Boxplots of raw amplicon reads of the libraries, which refer to sequence reads filtered by an exact primer or multiplex identifier sequence (MID) match, are shown in Figure 1. The median read count per amplicon in the presented study ranged between 214 and 439 with the exception of HLA-B exon 1, HLA-C exon 1, 7 and DRB exon 2. The short exon 1 and 7 amplicons with a median read count of 128, 162 and 124 were reduced due to a downscaled pooling scheme to compensate the higher PCR efficiency in the emulsion PCR. Likewise, the pooling factor for DRB exon 2 (mean 1,628) was increased to generate more reads because a number of loci are co-amplified using this generic primer set.

### Genotype analysis by Conexio ATF

**Figure 2:**
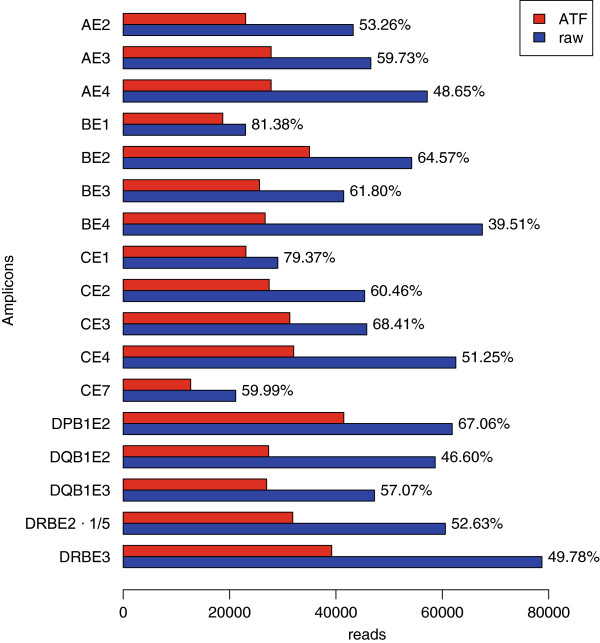
**Sequence read output after down-stream processing.** Representation of trimmed high quality reads generated by the GS Junior compared to the used sequence reads for allele assignment by the ATF software. Next to the bars percentage of ATF used reads are given.

For genotype assignment, the raw reads were imported via .fna file into the ATF software. Figure 2 gives an estimation of the continuous sequence loss from the imported raw reads up to the sequence reads used for the final allele assignment. The ATF software aligns the forward and reverse reads for each exon to the reference sequence in the “master layer” following automatic genotype assignment using an integrated allele database. Allele calling takes approximately 2–3 minutes (per GS run) using a standard workstation running Windows 7 (32 bit). Failed reads, derived from co-amplified genes (pseudogenes) or sequences with PCR or sequencing errors are sorted out by the software into the “failed layer” and these reads do not contribute to the final typing result. However, reads from the layers can be moved in both directions. In addition, the software allows deactivation and trimming of sequence reads generally used to exclude relatively rare sequences from the analysis.

### Allele assignment performance

From a total of 1,273 loci to be analysed we were able to generate 1,241 (97.3%) initial successful typing results using the novel NGS assay. Allele calling of 32 (2.7%) loci was impossible by insufficient read counts. NGS genotyping showed no mistyping compared to the Sanger sequencing results. In case of DRB3, we identified that the amplification of exon 2 in the groups DRB3*01 and *02 was inadequate leading to uncertain results (n = 20). The performance of the NGS HLA assay, broken down by number of typing per locus and accordance rates, is summarised in Table 2. Additionally, re-runs of the previously failed loci were successful. Typing results generated by the ATF software for HLA-A, -B, -C, -DRB1, -DQB1 and -DPB1 are listed in Additional file 1. The validation panel contained 89 (10.3%) homozygote genotypes and was compiled to cover almost all main groups across all loci. Moreover, the panel included rare alleles, null-alleles, not annotated genotypes and infrequent haplotypes to evaluate the clinical performance of the HLA assay. Two new alleles derived from samples of a proficiency program were correctly typed by NGS and showed the novel nucleotide variation in both sequencing directions with high coverage. In addition, the non-expressed alleles HLA-A*74:12 N (6 bp deletion in exon 3) and HLA-B*18:23 N (substitution in exon 3) were called properly by ATF. Out of 1,241 genotypes, 958 (77.2%) were called reliable without further editing. In 22.8% of the cases (mainly DRB results), manual editing of the NGS result was required. For instance, to identify HLA-C*03:04:01, only when in combination with HLA-C*03:03:01:01, it was necessary to move the sequence reads from the “failed layer” to the “master layer” in ATF software. This is a known issue described previously [15] and recently solved with the new ATF version 4.7. (see Additional file 2). Furthermore, sequencing of HLA-C*07 alleles showed normally a C homopolymer with 5 bases at the beginning of exon 4 but occasionally this stretch was not sequenced correctly in reverse direction. However, this position can be corrected by manual editing with high safety through existing error free forward sequence reads.

**Table 2:** HLA typing accuracy using NGS

**Locus**	**Total NGS typing**	**DNA not sufficient for NGS typing**	**No NGS result possible (insufficient read count)**	**Initial successfull NGS typing**	**Reference Sanger typing missing**	**Initial correct NGS typing compared to Sanger (if available)**	**NGS mistyping compared to Sanger**	**Concordance rate Sanger vs. NGS**
A	173	0	5	168 (97.1%)	1	167	0	100.0%
B	173	0	1	172 (99.4%)	0	172	0	100.0%
C	172	1	2	170 (98.8%)	4	166	0	100.0%
DRB1	172	1	2	170 (98.8%)	1	169	0	100.0%
DRB3	115	0	20	95 (82.6%)	4	91	0	100.0%
DRB4	79	1	1	78 (98.7%)	4	74	0	100.0%
DRB5	54	0	0	54 (100.0%)	1	53	0	100.0%
DQB1	162	11	0	162 (100.0%)	4	158	0	100.0%
DPB1	173	0	1	172 (99.4%)	105	67	0	100.0%
**all**	**1273**	**14**	**32**	**1241 (97.3%)**	**124**	**1117**	**0**	**100.0%**

Number of total NGS typings per locus is shown next to the number of initial successful NGS typings. A typing was initial successful if a result could be generated in the ATF software. Sanger typing results from the validation panel were available for all loci with few exceptions. An initial correct NGS typing could be only scored if a valid reference Sanger typing was available. No generated NGS result was wrong compared to the reference typing.

In general, DRB1 genotyping was mainly conclusive with the exception of some challenging homozygote samples. In these rare cases manual editing was needed by incorrect allocation of a low number of sequence reads of co-amplified pseudogenes (DRB2, DRB6, DRB7 and DRB9) or PCR artefacts formed through in vitro recombination between DRB1 and DRB3/4/5. These recently described PCR crossover sequences [24] can only be seen with technologies using high sequencing depths. Some recombinant sequences correspond to annotated alleles in the IMGT/HLA database and it is still not clear if the assigned sequences are PCR artefacts or true alleles. Nevertheless, the DRB1 locus can be analysed successfully by deactivation of such sequence reads identified by DRB3/4/5 specific sequence motifs.

### Reduction of HLA ambiguity by NGS

**Figure 3:**
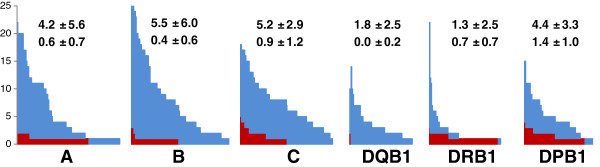
**Residual ambiguities of HLA-A, -B, -C, -DQB1, -DRB1 and -DPB1.** Ambiguities for the Sanger typing method are given in blue, red bars represent the number of ambiguities of the Genome Sequencer typing method, e.g. samples with 20 ambiguities for Sanger typing in locus A have 2 ambiguities with GS typing. Mean and standard deviation are given per locus, the first line represents the ambiguities from Sanger typing, the second line from GS typing.

**Figure 4:**
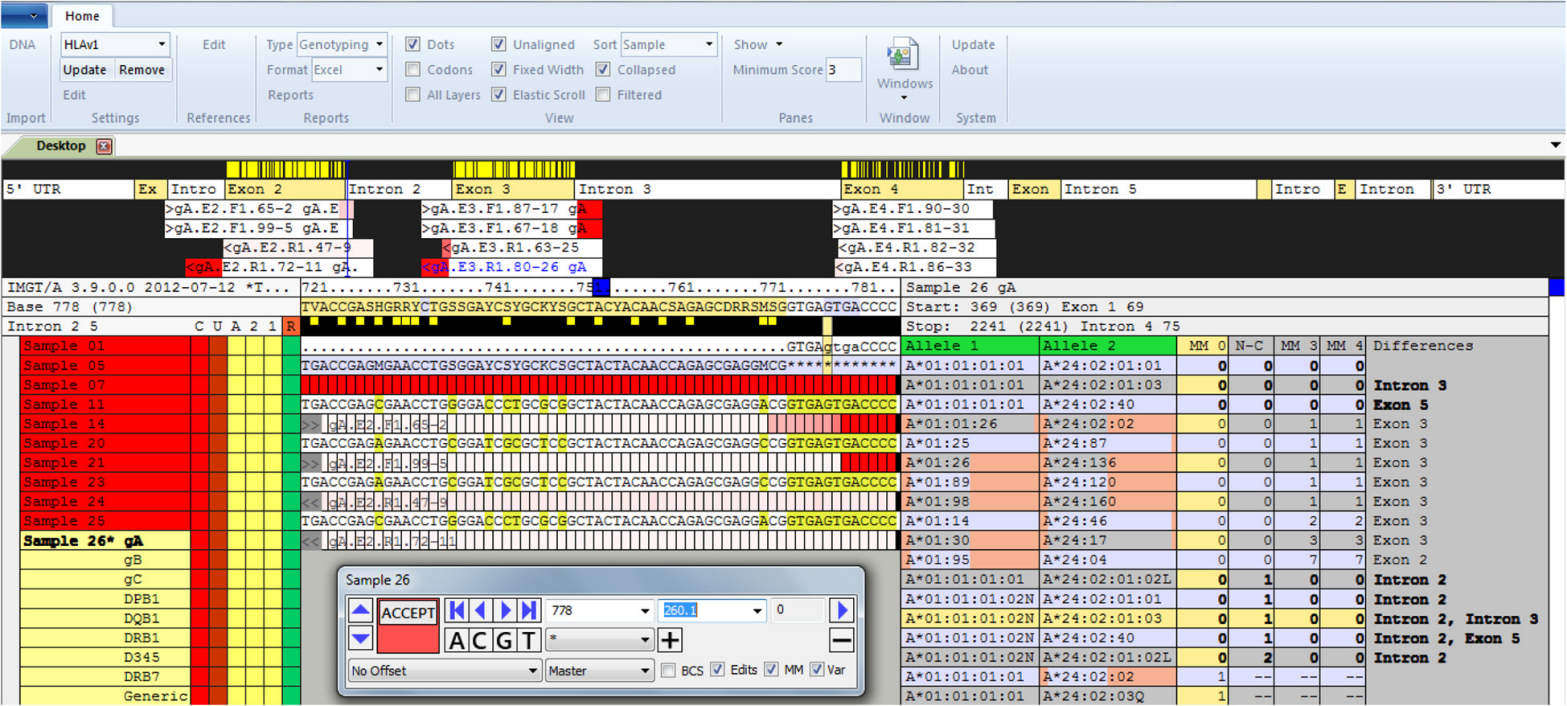
**ATF 4.7 user interface.** HLA-A genotype assignment showing the “master layer”. The forward and reverse sequence reads are aligned to exon 2, 3 and 4 with partial coverage of intronic regions. Read counts and sequencing direction are displayed in white bars. For instance, exon 4 is represented by two (allele 1 and 2) forward sequences with coverage of 90, respectively 81 sequence reads and two reverse sequences including 82 and 86 sequence reads. Underneath the exon map, the consensus sequence and the genotype assignment are shown. The phased sequences at the exon 2/intron 2 boundary are illustrated and propose the combination HLA-A*01:01:01:01, 24:02:01:01 with zero mismatches (master layer MM) compared to the database. The non- expressed A*01:01:01:02 N and the low expressed A*24:02:01:02 L are excluded by intron 2 variations as shown in the non coding (N-C) column. Mismatches in the phase layer 3 and 4 (MM3, MM4) demonstrate cis/trans ambiguities that would arise if the phase of SNPs in exon 3 and exon 2 cannot be defined (generic Sanger sequencing). The remaining alleles A*24:02:01:03 (variation in intron 3) and A*24:02:40 (synonymous substitution in exon 5) cannot be excluded and are therefore displayed likewise with zero mismatches.

To estimate the ambiguity resolution of this newly developed GS HLA assay, we defined ambiguity strings quantitatively at the 4 digit level (including non-expressed alleles) of Sanger sequencing results versus clonal NGS results. The mean ambiguity reduction for the analysed loci was 93.5%. Numbers of ambiguities per locus and typing strategy are given in Figure 3, except DRB3/4/5. There was no significant improvement for DRB3, DRB4 and DRB5 typing compared to Sanger sequencing. The highest ambiguity reduction was found for HLA-DQB1 (100%) and HLA-B (92.7%). Additionally, for HLA-A and HLA-C the ambiguities were greatly reduced by about 85.7%, and 82.7% respectively, using clonal sequencing. The loci DRB1 (46.1%) and DPB1 (68.2%) ranked at the end with a slightly reduced benefit concerning ambiguity reduction justified by a group-specific Sanger-based DRB1 approach and the lack of exon 3 sequence information for DPB1 in both assays. To meet the defined EFI standards for high-resolution typing, we re-analysed selected samples with a new release of the ATF Software (version 4.7 provided for beta testing) enabling the analysis of introns to exclude some more null alleles (Figure 4). The new version made it possible to rule out the following alleles: HLA-A*01:01:01:02 N (intron 2), HLA-A*03:01:01:02 N (intron 4), HLA-29:01:01:02 N (intron 4) and HLA-B*15:01:01:02 N (intron 1). A total of 243 out of 245 non-expressed variants (DRB3/4/5 excluded) can be currently (IMGT 3.7.0) identified using this novel NGS HLA assay. Exon 1 typing of HLA-A is not implemented at the moment due to a reduced frequency of A*68 alleles (exactly A*68:01:02) in our population; therefore we cannot exclude HLA-A*68:11 N. Furthermore, a single base substitution at the end of exon 2 leading to DRB1*03:68 N is located at the 3’ primer binding site of the generic reverse primer of DRB, so this null allele is not excludable. These both non-expressed and clinical significant alleles must be addressed by Sanger sequencing during verification typing.

## Discussion

Recently introduced next-generation sequencing platforms have the potential to facilitate high-resolution HLA genotyping in the forthcoming period through a general reduction of phase ambiguities, while extending the regions to be sequenced. Using the sequencing capacity of these technologies, it is possible to sequence the entire major histocompatibility complex (MHC) [25] in order to discover hidden transplantation determinants. On the other hand, in the near future, sequencing of the total human exome could become a state-of-the-art technique for deciphering genetic variations affecting the outcome of HSCT. However, at this time, exclusively classical transplantation antigens are defined routinely in accordance with the applicable requirements provided by the associated societies. So far, the definition of HLA-A, -B, -C, (exon 2, 3) -DRB1 (exon 2) and -DQB1 (exon 2, 3) at 4-digit level is currently used for related and unrelated donor selection. The clinical relevance of using sequence information outside the peptide binding site, or at higher resolution (6- and 8-digit) in terms of alloreactivity still remains unknown except in variations in the coding or non-coding regions leading to null alleles characterised by a lack of HLA expression on the cell surface [26]. To exclude non-expressed alleles, extended typing of HLA-A (exon 1, 4 and intron 2, 4), HLA-B (exon 1, 4 and intron 1) and HLA-C (exon 1, 4, 7) is necessary and effectively demonstrated in this study.

The advantage of this GS HLA typing assay compared to recently published shotgun protocols relies on scalability through the use of relevant exon sequence information, therefore saving sequencing capacity. Analysing large or full genomic regions of each HLA gene will flood the IMGT/HLA database in the near future and the only argument for it is that it has the ability to discover very rare null alleles. An attempt to completely analyse non-coding regions of HLA alleles would be challenging not only due to missing intronic sequences of the broad majority of the annotated alleles, but because it would be disproportionate to the intended objective. Nevertheless, the discussion remains ongoing.

Applying the GS Junior a large number of sequence reads did not pass quality criteria and was rejected by the system, rendering it unusable. Similarly, approximately 35% of the raw reads did not pass the ATF software due to errors. Consequently, a substantial sequence loss has to be taken into account. Using this exon-based sequencing approach, it is extremely important to avoid imbalanced conditions between exons. Underrepresentation of sequences is not a relevant problem in our assay and is usually solved by introducing a scaling factor at the amplicon pooling step. Interestingly, using shotgun sequencing variability in read depth across the region is an issue affected by the fragmentation process and read length [21,27]. Generally, an allele dropout due to preferential amplification refers to all currently available protocols and is ruled out by PCR optimisation and extensive validation, as shown in this work. Most notably, long-range PCR might be more susceptible to allele dropout. Targeting many HLA regions by multiple PCRs enhances safety by identifying heterozygous positions, although fusion primer strategies require increased MID validation efforts. In conclusion, inadequate sequence coverage is a well-known reason for dropout of alleles (sequences). In this study the average read count was 182 per amplicon, leading to reliable typing results and reasonable costs per sample. Coverage is an important factor and must therefore be specified carefully based on validation data.

Moreover, reliable typing results depend on DNA quality, which is still a critical factor in routine laboratories. The use of RNA for the definition of HLA loci reduces the library construction dramatically, but clinical samples are often not suitable for cDNA preparation and the exclusion of null alleles characterised by intonic variations is not possible. The problem likewise arises when using long-range PCR requiring intact, high-quality genomic DNA for secure amplification. For clinical laboratories, the complex workflow is a major drawback as it often allows for human errors. For instance, sample mix-up during PCR setup or library processing was successfully solved in this study through the automation of the workflow. Further issues, including occasionally observed sequence-specific errors - also identified in non-homopolymer motifs - are still quite difficult to handle. Thus, it is mandatory to sequence in forward and reverse direction when using NGS, similarly as implemented in generic Sanger sequencing.

454 sequencing is reaching the longest reads length to date and we demonstrated that we were able to obtain phased exon sequence information from nearly all selected regions. Generally, phase ambiguities rise frequently in conserved regions or sequences with a reduced incidence of widely spaced SNPs and cannot be excluded by the currently developed methods. In our case, phase definition across 425 bp of each single read is feasible. Ambiguities may occur when the phase between exons cannot be defined, based on HLA alleles generated by exon shuffling. However, these exemptions are indeed rare and could not be identified in our validation panel. By implementing the new ATF 4.7 version phasing is further improved by inclusion of polymorphic sites located in the intron. As expected, new developments in this field are still ongoing and already realised by a sequencing length of 700 bp using the FLX + version. New protocols reaching very long reads will be available in the near future even for the GS Junior.

Due to the use of liquid handling robots, we were able to type six HLA loci of ten samples within 2.5 days with the GS Junior. Using a reduced primer panel (eight amplicons) to reach the minimal requirements for high-resolution typing, frequently termed as intermediate resolution typing, increases the throughput to twenty samples per GS Junior run. This application is ideally suitable for registry typing, cordblood typing or donor search in families, but also more cost-effective than traditional low-resolution typing techniques used in our laboratory. By the use of the GS FLX we are able to scale-up the throughput to 40, respectively 80 samples per run using a four region gasket.

## Conclusions

A typical tissue typing laboratory needs to be flexible, providing HLA typing results within a short turnaround time and notably without fail. From this perspective we developed, validated and implemented a next-generation sequencing HLA approach using 454 sequencing. Our primary aim was to eliminate the major outstanding obstacles in the NGS workflow to ensure a smooth daily typing routine. The presented study demonstrates that this novel HLA NGS assay performs with 100% reliability and is particularly suitable for unrelated donor HLA typing, according to the policies of the EFI. The workflow fits very well in medium- and high-throughput typing laboratories and could become attractive for donor registries. NGS seems to become one of the new methods in the field of Histocompatibility and Immunogenetics.

## Methods

### Validation panel and reference method

In this study we analysed 173 samples including patients and donors from our transplant programs (n = 125) and samples from two external proficiency typing programs (DZA German Cell Exchange/ CET Central European proficiency testing) (n = 48). The validation panel was well composed using samples processed with different DNA isolation techniques, cordblood, samples from treated patients and the associated unrelated and related donors. To evaluate the performance of the developed assay, we typed also rare alleles, null-alleles, still not described alleles and samples with infrequent haplotypes. Each clinical specimen was obtained after subjects signed a written informed consent. The samples been previously typed for HLA-A and B including exons 2–4 (AlleleSEQR HLA-A/B, Abbott Molecular, Des Plaines, IL), HLA-C and DQB1 including exons 2–3 (S3 HLA-C/DQB1; Protrans, Hockenheim, Germany) and exon 2 of HLA-DPB1 (Conexio Genomics, Fremantle, Australia) according to the manufacturer’s instructions. HLA-DRB1 (exon 2) was sequenced group-specific as described previously [28]. The sequencing device was a 3130×l Genetic Analyzer (Life Technologies, Carlsbad, CA). Electropherograms were analysed by the Assign 3.5+ software (Conexio Genomics) using the IMGT/HLA database release 3.7.0.

### DNA isolation

Genomic DNA was isolated from EDTA-blood using the MagNaPure Compact Nucleic Acid Isolation Kit - Large Volume (Roche Diagnostics, Mannheim, Germany) or the Nucleon genomic DNA extraction kit (VWR International, Wien, Austria) according to the manufacturer’s instructions. DNA quality and concentration were determined photometrically (BioPhotometer, Eppendorf, Hamburg, Germany) and diluted with PCR grade water to a concentration of 10 ng/μl.

### Primer design

Based on the fusion primer concept [24], including adapter sequence and a 10 bp multiplex identifier sequence (see Additional file 3), we designed locus specific amplification primers targeting overall 17 exons of HLA-A, -B, -C, -DRB, -DQB1 and -DPB1. Primer specificity, genomic position and amplicon length are given in Table 3. Complete coverage was achieved for nearly all exons except DRB and DQB1. The 5’ primer and the 3’ primer for HLA-DQB1 exon 2 extend 9, respectively 2 bases in the coding region. The 3’ DRB primer extend 20 bases into the conserved region of the exon 2. In addition, the DRB systems (exon 2, 3) showed a reduced specificity, which allowed us to co-amplify DRB3, DRB4 and DRB5 (DRB3/4/5) but also some undesired pseudogenes of the DRB locus. Primers were synthesized and desalted by Metabion GmbH (Martinsried, Germany).

**Table 3:** PCR primer and pooling factor information for the GS Junior HLA typing assay

**Locus**	**Exon**	**Reference**	**Forward primer**		**Reverse primer**		**Amplicon length**	**Pool**	**Pooling Factor**
			**Region**	**Position**	**Region**	**Position**			
A	2	A*01:01:01:01	Exon 1	51-67	Intron 2	66-83	576	long	1
A	3	A*01:01:01:01	Intron 2	150-171	Intron 3	65-83	521	long	1
A	4	A*01:01:01:01	Intron 3	539-559	Intron 4	76-91	541	long	1
B	1	B*07:02:01	5’NCR	4998-5018	Intron 1	45-59	278	short	0.5
B	2	B*07:02:01	Intron 1	45-59	Intron 2	25-43	467	long	1
B	3	B*07:02:01	Intron 2	181-195	Intron 3	36-52	462	long	1
B	4	B*07:02:01	Intron 3	501-519	Exon 5	27-45	558	long	1
C	1	C*01:02:01	5’NCR	210-230	Intron 1	42-62	279	short	0.5
C	2	C*01:02:01	Intron 1	42-62	Intron 2	42-58	487	long	1
C	3	C*01:02:01	Intron 2	145-165	Intron 3	65-83	531	long	1
C	4	C*01:02:01	Intron 3	444-461	Intron 4	76-94	584	long	1
C	7	C*01:02:01	Intron 6	62-80	Exon 8	14-33	366	short	0.5
DRB	2	DRB1*01:01:01	Intron 1	5168-5189	Exon 2	250-270	545	long	6
DRB	3	DRB1*01:01:01	Intron 2	2143-2163	Intron 3	1-20	459	long	1
DQB1	2	DQB1*02:01:01	Intron 1 | Exon 2	1429-1438 | 1-9	Exon 2 | Intron 2	268-270 | 1-16	356	short	0.5
DQB1	3	DQB1*02:01:01	Exon 3	43-62	Intron 3	1-27	357	short	0.5
DPB1	2	DPB1*03:01:01	Intron 1	4490-4517	Intron 2	3-27	408	long	1

### PCR amplification

The polymorphic regions of HLA-A exon 2–4, HLA-B exon 1–4, HLA-C exon 1–4, 7 and HLA-DRB, HLA-DQB1 exon 2–3 as well DPB1 exon 2 were amplified in 20 μl reactions using 96 well FAST plates (Life Technologies). The PCR mixture consisted of 1× reaction buffer 2 (with 1.5 mM Mg^++^), 200 μM dNTPs, 0.88U Expand High Fidelity PCR enzyme (Roche Diagnostics), 2× PCR Enhancer (Biozyme, Blaenavon, UK), primer at 0.5 μM each, and 50 ng of genomic DNA. PCR amplification was carried out in a Veriti Thermal Cycler (Life Technologies) using the subsequent PCR protocol: initial denaturation for 2 minutes at 95°C followed by 37 cycles consisting of 30 seconds at 95°C, 30 seconds at 59°C, 1 minute at 72°C and a final extension for 7 minutes at 72°C.

### Amplicon cleanup, quantification, normalization, and pooling

For purification of the PCR products, quantification of amplicons and equimolar pooling, commonly referred to as library preparation, was done on a Microlab STAR liquid handling robot (Hamilton Robotics, Martinsried, Germany). The process is illustrated in an application note available from Hamilton (Lit. No. MR-1107-03/00). Unspecific PCR products were removed by Agencourt AMPure XP beads (Beckman Coulter, Vienna, Austria) using a one to one DNA per bead ratio. Purified amplicons were quantitated by Pico-Green (Life Technologies) utilizing an external Infinite 200Pro fluorometer (Tecan, Grödig, Austria) using the Magellan v7.0 Software (Tecan). Based on the standard concentrations, the signals were directly translated to ng/μl in an .xls file and electronically provided for the pooling process, an additional report with quality control checks in .pdf format is generated. The coefficient of determination (validation criteria r^2^ > 0.98) was calculated from eight DNA standards in a range from 0-100 ng/μl. For equimolar normalisation of the 176 PCR products, the concentrations from the amplicons were converted in molecules/μl using a configuration file with associated amplicon lengths. Due to the higher PCR efficiency of short amplicons and the fact that the DRB-specific primer system amplifies additional pseudogenes, we optimized the pooling of the products to improve the read balancing post sequencing. Similarly, we separated short and long amplicons from each other in the emulsion PCR to reach identical conditions. The allocation of the amplicons and the pooling factor are shown in Table 1. Two pools, the first (short pool) containing the 5 shortest amplicons and the second pool (long pool) with the remaining 12 amplicons, were automatically generated from each amplicon with 1 × 10^9^ molecules. The amplicon library was diluted to 5 × 10^5^ molecules and again manually purified by AMPure XP beads (Beckman Coulter).

### Emulsion PCR, bead enrichment, and sequencing

Emulsion PCR, breaking, and bead enrichment were carried out using the GS Junior Titanium emPCR Kit Lib-A, emPCR Reagents Lib-A kit, Oil and Breaking Kit and the Bead Recovery Reagents Kit according to the supplier’s instructions (Roche Diagnostics). For the emulsion PCR we used a copy-per-bead ratio of 0.75 for the short pool, respectively 0.5 for the long pool. Bead enrichment was done with the robotic enrichment module (Roche Diagnostics) on a Microlab STARlet robot (Hamilton Robotics). After DNA capture bead enrichment, to remove non-DNA carrying beads, automated bead counting was performed using Scepter 2.0 (Merck Millipore, Billerica, MA). Finally, we loaded 600,000 beads onto the PicoTiterPlate (PTP) (Roche Diagnostics). Sequencing was carried out according to standard Roche/454 protocols using the GS Titanium Sequencing Kit (Roche Diagnostics) and the GS Junior device.

### Data analysis

DNA sequence reads were obtained in FASTA format (.fna). Using NGS technologies, it is a commonly accepted observation that the longer reads become, the more prone they may be to errors [29]. In a preprocessing step with an offset to enhance similarity, high quality reads were trimmed after the targeted exon at the exon/intron boundary. Therefore, a post analysis script was defined in the xml settings of the GS Junior data processing. This xml script triggers a Perl script (The Perl Foundation, Walnut, CA, USA) performing the preprocessing step first. Trimming points were calculated by summing lengths of MID, primer and exon and the distance of the primer to the exon. The used trimming approach ensures that all exon information is kept and allows for higher readcounts in the allele assignment software ATF version 1.1.0.33 (Conexio Genomics). Subsequently, the post analysis script adds marker sequences to the data files intrinsically to each specific sequencing run and its corresponding MID sample association file to prevent mix-up of different runs. For every sequencing run a detailed report (.pdf format) is generated using a Java-written module summarising most important run performance parameters and quality information (see Additional file 4). Automated preprocessing takes place on the GS Junior attendant PC running Linux CentOS 5.4, accordingly the post analysis script transfers sequencing and report files to the laboratory server. The processed .fna files were imported into the ATF software (Conexio Genomics) for genotype assignment using the IMGT-HLA database release 3.7.0.

### Sample tracking

After adding the sample barcodes to the fully documenting MS Excel run protocol, formatted work lists for PCR setup, purification, quantification and pooling as well as a MID-sample association file for ATF software were automatically generated by Excel Macros (VBA). To avoid loading wrong accessories or samples in the automated process, all components were equipped with barcodes and every sample is tracked by the liquid handling robots, allowing for LIMS integration. The automated amplicon library preparation was subdivided into three modules to enable an individual or continuous run.

### LIMS import

**Figure 5:**
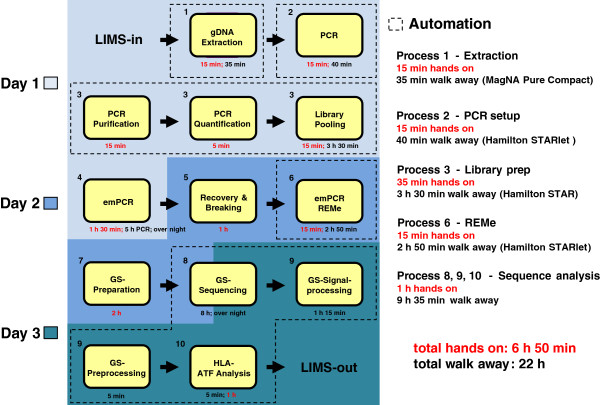
Schematic illustration of the HLA typing workflow for ten samples.

After genotype assignment a MS Excel report was created by ATF and loaded into HLAcombine, an in-house software product written in C# to combine typing results from different assays and reducing alleles to four digit results. With an xml interface results were imported into LIMS (hlaSYSTEM, AVALAS GmbH, Nordhausen, Germany) and queued for medical validation. The entire workflow, including run-time and hands-on time, is illustrated in Figure 5.

## Competing interests

The authors declare no competing interests.

## Authors’ contributions

MD designed the HLA sequencing assay, analysed data and wrote the manuscript. NN designed and developed software for the analysis pipeline, performed bioinformatics including data analysis and figure preparation, and critically reviewed the manuscript. SS, KH designed the HLA sequencing protocol and helped to automate the entire workflow. JP provided intellectual support for the sequencing protocol and reviewed the manuscript. CS, ER performed the validation study and collected data from the reference method. HP reviewed the manuscript. CG designed research and critically reviewed the manuscript. All authors read and approved the final manuscript.

## Supplementary Material

Additional file 1 NGS genotyping results of 173 sample. Click here for file

Additional file 2 ATF 4.7 interface and HLA-C*03:03:01, 03:04:01:01 assignment. Click here for file

Additional file 3 MID sequence information. Click here for file

Additional file 4 Run performance quality report. Click here for file
