# Supplementary material for: Rapid, scalable and highly automated HLA genotyping using next-generation sequencing: a transition from research to diagnostics
Source: BMC Genomics. 2013 Apr 4;14:221. doi: 10.1186/1471-2164-14-221 (PMC3639865; doi:10.1186/1471-2164-14-221)

Additional file 2 – ATF 4.7 interface and HLA-C*03:03:01, 03:04:01:01 assignment.
Sequence reads are aligned to exon 1, 2, 3, 4 and 7. The only difference between HLA-C*03:03:01 and C*03:04:01:01 is located at the end of exon 2. The inability to call this genotype with the older ATF version was automatically solved by ATF 4.7. The blue bar indicate the variation (nt756 R=A+G) at the last position of exon 2.

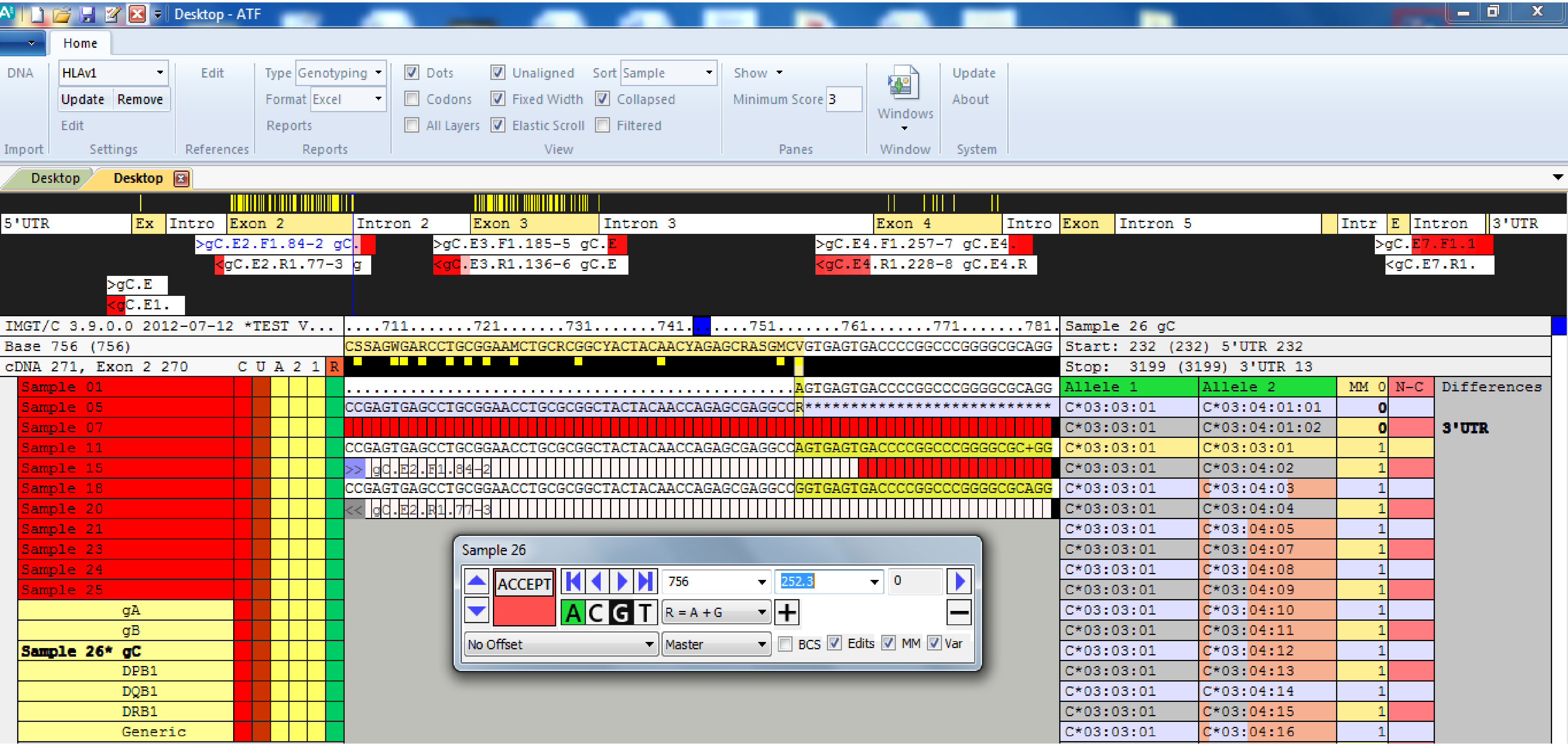

Supplement: Additional file 2 — ATF 4.7 interface and HLA-C*03:03:01, 03:04:01:01 assignment. [file 1471-2164-14-221-S2.pdf]
