# Supplementary material for: Rapid, scalable and highly automated HLA genotyping using next-generation sequencing: a transition from research to diagnostics
Source: BMC Genomics. 2013 Apr 4;14:221. doi: 10.1186/1471-2164-14-221 (PMC3639865; doi:10.1186/1471-2164-14-221)
Supplement: Additional file 4 — Run performance quality report. [file 1471-2164-14-221-S4.pdf]

tiGSj048-2011d

Sequencing Run		Run Processor Results	
Name	tiGSj048-2011d	Run date	2011/12/30 10:01:34
Regions	1	Total Bases Passed Filter	36,891,809
Cycles	200	Passed Filter Wells	79,050

Library Region	1	Median	Total
Raw Wells	179,605	179,605	179,605
Key Pass Wells	170,345	170,345	170,345
Median ReadLength	494.00		494.00
Passed Filter Wells	79,050	79,050	79,050
% Dot + Mixed	9.60	9.60	9.60
% Short	43.99	43.99	43.99
% Passed Filter	46.41	46.41	46.41

Control Sequences Region		1	Median	Total
Raw Wells		179,605	179,605	179,605
All ATGC Wells		3,925	3,925	3,925
All CATG Wells		2,813	2,813	2,813
%Match	400 bp, 100%	49.66	49.66	49.66
	>=98%	81.73	81.73	81.73
	>=95%	88.71	88.71	88.71

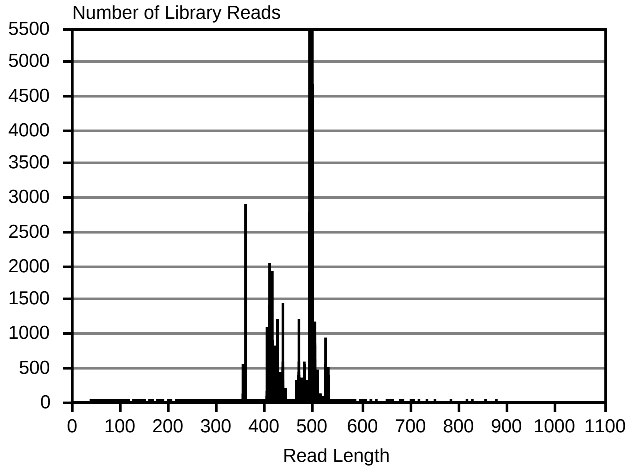

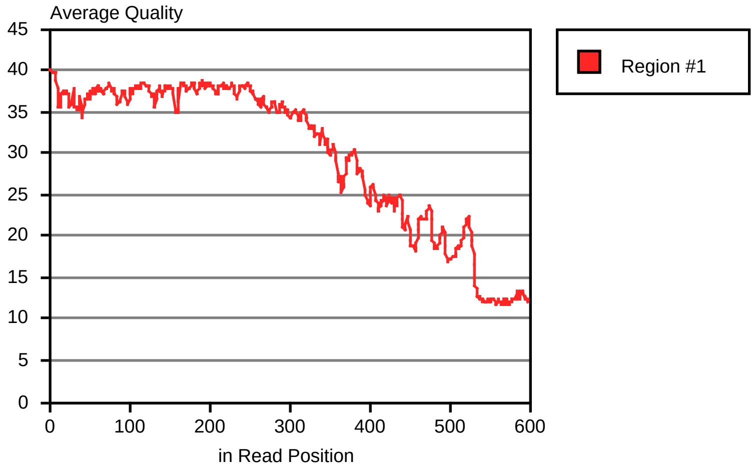

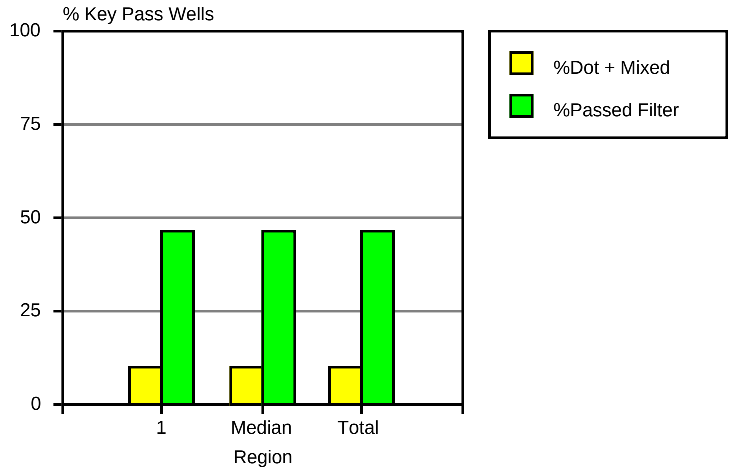
% Key Pass Wells
100
75
50
25
0
1
Median
Total
Region
%Dot + Mixed
%Passed Filter

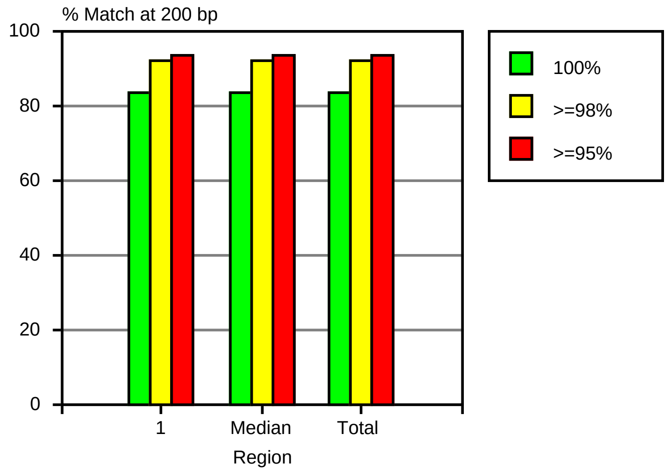
% Match at 200 bp
100
80
60
40
20
0
1
Median
Total
Region
100%
>=98%
>=95%

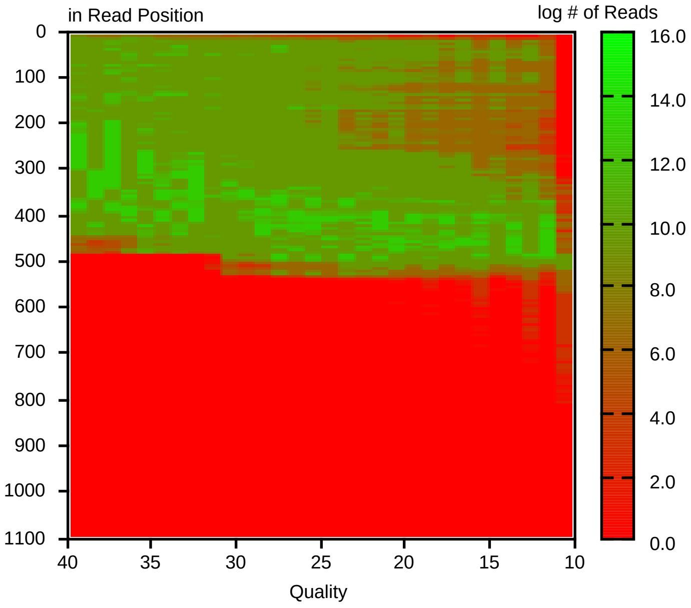
in Read Position
log # of Reads
0
100
200
300
400
500
600
700
800
900
1000
1100
40
35
30
25
20
15
10
Quality
16.0
14.0
12.0
10.0
8.0
6.0
4.0
2.0
0.0

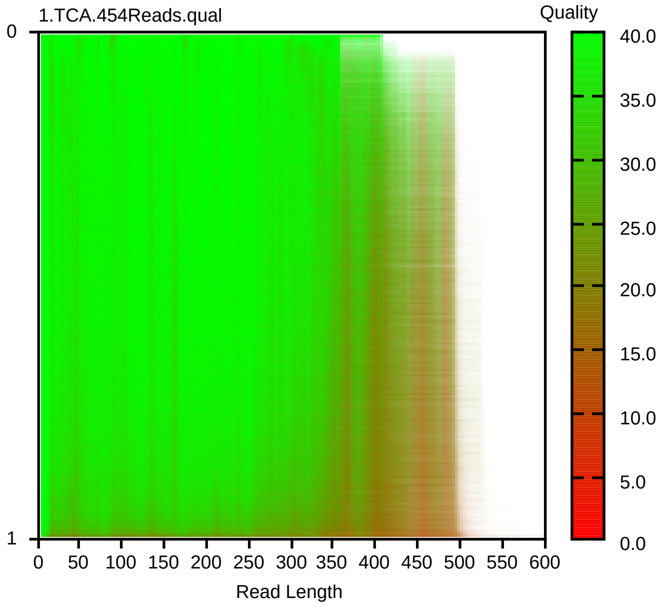
1.TCA.454Reads.qual
Quality
0
1
0
50
100
150
200
250
300
350
400
450
500
550
600
Read Length
40.0
35.0
30.0
25.0
20.0
15.0
10.0
5.0
0.0
